# Protist predation promotes antimicrobial resistance spread through antagonistic microbiome interactions

**DOI:** 10.1093/ismejo/wrae169

**Published:** 2024-09-04

**Authors:** Chen Liu, Yijin Wang, Zeyuan Zhou, Shimei Wang, Zhong Wei, Mohammadhossein Ravanbakhsh, Qirong Shen, Wu Xiong, George A Kowalchuk, Alexandre Jousset

**Affiliations:** Jiangsu Provincial Key Laboratory for Solid Organic Waste Utilization, Key Laboratory of Organic-Based Fertilizers of China, Jiangsu Collaborative Innovation Center for Solid Organic Wastes, Educational Ministry Engineering Center of Resource-Saving Fertilizers, Nanjing Agricultural University, No. 1 Weigang, Xuanwu district, Nanjing 210095, People’s Republic of China; Jiangsu Provincial Key Laboratory for Solid Organic Waste Utilization, Key Laboratory of Organic-Based Fertilizers of China, Jiangsu Collaborative Innovation Center for Solid Organic Wastes, Educational Ministry Engineering Center of Resource-Saving Fertilizers, Nanjing Agricultural University, No. 1 Weigang, Xuanwu district, Nanjing 210095, People’s Republic of China; Jiangsu Provincial Key Laboratory for Solid Organic Waste Utilization, Key Laboratory of Organic-Based Fertilizers of China, Jiangsu Collaborative Innovation Center for Solid Organic Wastes, Educational Ministry Engineering Center of Resource-Saving Fertilizers, Nanjing Agricultural University, No. 1 Weigang, Xuanwu district, Nanjing 210095, People’s Republic of China; Jiangsu Provincial Key Laboratory for Solid Organic Waste Utilization, Key Laboratory of Organic-Based Fertilizers of China, Jiangsu Collaborative Innovation Center for Solid Organic Wastes, Educational Ministry Engineering Center of Resource-Saving Fertilizers, Nanjing Agricultural University, No. 1 Weigang, Xuanwu district, Nanjing 210095, People’s Republic of China; Jiangsu Provincial Key Laboratory for Solid Organic Waste Utilization, Key Laboratory of Organic-Based Fertilizers of China, Jiangsu Collaborative Innovation Center for Solid Organic Wastes, Educational Ministry Engineering Center of Resource-Saving Fertilizers, Nanjing Agricultural University, No. 1 Weigang, Xuanwu district, Nanjing 210095, People’s Republic of China; Ecology and Biodiversity Group, Department of Biology, Institute of Environmental Biology, Utrecht University, Padualaan 8, 3584 CH Utrecht, The Netherlands; Jiangsu Provincial Key Laboratory for Solid Organic Waste Utilization, Key Laboratory of Organic-Based Fertilizers of China, Jiangsu Collaborative Innovation Center for Solid Organic Wastes, Educational Ministry Engineering Center of Resource-Saving Fertilizers, Nanjing Agricultural University, No. 1 Weigang, Xuanwu district, Nanjing 210095, People’s Republic of China; Jiangsu Provincial Key Laboratory for Solid Organic Waste Utilization, Key Laboratory of Organic-Based Fertilizers of China, Jiangsu Collaborative Innovation Center for Solid Organic Wastes, Educational Ministry Engineering Center of Resource-Saving Fertilizers, Nanjing Agricultural University, No. 1 Weigang, Xuanwu district, Nanjing 210095, People’s Republic of China; Ecology and Biodiversity Group, Department of Biology, Institute of Environmental Biology, Utrecht University, Padualaan 8, 3584 CH Utrecht, The Netherlands; Jiangsu Provincial Key Laboratory for Solid Organic Waste Utilization, Key Laboratory of Organic-Based Fertilizers of China, Jiangsu Collaborative Innovation Center for Solid Organic Wastes, Educational Ministry Engineering Center of Resource-Saving Fertilizers, Nanjing Agricultural University, No. 1 Weigang, Xuanwu district, Nanjing 210095, People’s Republic of China; Ecology and Biodiversity Group, Department of Biology, Institute of Environmental Biology, Utrecht University, Padualaan 8, 3584 CH Utrecht, The Netherlands

**Keywords:** protist predation, microbial interactions, antibiotic resistance genes, soil resistome

## Abstract

Antibiotic resistance has grown into a major public health threat. In this study, we reveal predation by protists as an overlooked driver of antibiotic resistance dissemination in the soil microbiome. While previous studies have primarily focused on the distribution of antibiotic resistance genes, our work sheds light on the pivotal role of soil protists in shaping antibiotic resistance dynamics. Using a combination of metagenomics and controlled experiments in this study, we demonstrate that protists cause an increase in antibiotic resistance. We mechanistically link this increase to a fostering of antimicrobial activity in the microbiome. Protist predation gives a competitive edge to bacteria capable of producing antagonistic secondary metabolites, which secondary metabolites promote in turn antibiotic-resistant bacteria. This study provides insights into the complex interplay between protists and soil microbiomes in regulating antibiotic resistance dynamics. This study highlights the importance of top–down control on the spread of antibiotic resistance and directly connects it to cross-kingdom interactions within the microbiome. Managing protist communities may become an important tool to control outbreaks of antibiotic resistance in the environment.

## Introduction

The accumulation and dissemination of antibiotic resistance in the environment pose a global menace to human health and are recognized as one of the major global and local health challenges [1, 2]. Soil, in particular, serves as a reservoir of antibiotic resistance genes (ARGs) [3]. Addressing the rise of antibiotic resistance in environments such as soil necessitates a better understanding of the drivers underlying the emergence and accumulation of ARGs, as well as antibiotic-resistant microorganisms [1, 4].

Historically, the rise of antibiotic resistance in soil has been linked to anthropogenic factors such as the introduction of antibiotics from wastewater and manure [2, 5, 6]. However, antibiotic resistance also occurs in natural or undisturbed soils, independent of human influence [7], and predates the use of synthetic antibiotics by humans [8–10]. Thus, the functioning of ecosystems may contribute to the spread and emergence of both native and exogenous ARGs. For instance, many soil microorganisms produce antimicrobial compounds to inhibit competitors, and a certain level of antibiotic resistance is often a component of microbial fitness in ecosystems [11, 12].

Protistan predators, though often overlooked, are crucial components of soil microbiomes and are considered primary predators of soil bacteria [13]. Protist predation represents a significant evolutionary pressure on bacteria, influencing bacterial diversification and community succession. For example, phagotrophic protists utilize toxic metals to kill ingested bacteria and causally pose a strong selection for bacterial copper resistance, which leads to the accumulation of copper–resistant bacterial lineages [14, 15]. Although protists have been associated with higher levels of antibiotic resistance in soils [16], the underlying mechanisms and causality remain poorly understood. Bacterial antibiotic resistance itself is not yet known to have any direct impact on protist predation. Yet, protists have been reported to select secondary metabolite-producing species over non-toxic competitors [17] and stimulate bacterial antibiotic production [18]. Therefore, we hypothesize that protists may indirectly cause higher levels of antibiotic resistance by fostering the production of antimicrobial compounds by bacteria.

In this study, we aim to investigate the role of protists as drivers of antibiotic resistance. Our hypothesis posits two main points: (i) the abundance of ARGs increases with the relative abundance of soil protists, and (ii) this increase is mediated by co-associated life-history traits of bacteria, particularly their capacity to produce antibiotics, rather than antibiotic resistance per se. To test our hypothesis, we conducted metagenomic analysis on 6-week field soils to assess the influence of soil protists on ARGs and soil bacteria. We generated metagenome-assembled genomes (MAGs) from metagenomic sequencing data, which allowed us to identify potential ARGs and biosynthetic gene clusters (BGCs) encoding putative antibiotic metabolites within these genomes. Laboratory experiments were then conducted to co-culture bacterial communities with protist predators and bacteria that produce antibiotic secondary metabolites. We then used metatranscriptomics data to evaluate the functional response of bacteria to protist predation. Overall, our research aims to elucidate microbial mechanisms underlying protist-induced increases in ARG abundances.

## Materials and methods

### Experimental design and collection of soil samples

To track the dynamics of both soil microbial communities and resistomes, we used a rhizobox sampling system that has been documented in previous publications [19, 20]. The semi-natural rhizobox sampling system was employed within a tomato field soil, allowing repeated sampling of identical tomato plants within a single cropping season by removing individual nylon mesh bags from the central layer of the rhizobox. In short, tomato seedlings were incubated in the greenhouse before planting in the field within rhizoboxes. The rhizobox field experiment was conducted in Qilin, Nanjing, China, during the late spring cropping season. We collected soil samples at week 0, 3, 4, 5, and 6 for each tomato plant (in total, 8 tomato plants were selected). Deoxyribonucleic acid (DNA) extraction from 0.5 g of soil was performed utilizing the PowerSoil™ DNA Isolation Kit (Mo Bio Laboratories Inc., Carlsbad, CA, United States) following manufacturer’s instructions. DNA extracts were quantified using a NanoDrop spectrophotometer (ND2000, Thermo Scientific, DE, United States). Aliquots of DNA extract were used for quantitative polymerase chain reaction (qPCR) analyses, and the other was used for metagenomic shotgun sequencing.

### Quantitative polymerase chain reaction assay

To compare the relationships between copy numbers and estimated reads numbers by metagenomics of 16S ribosomal ribonucleic acid (rRNA) genes targeting soil prokaryotes, we first determined the total copy numbers of 16S rRNA genes targeting soil prokaryotes using qPCR with primer sets Eub338/Eub518 [21] and the SYBR Premix Ex Taq Kit (Takara, Dalian, China) following the manufacturer’s instructions. Measurements were carried out in triplicate for each sample on a 7500 Fast Real-Time PCR System (Applied Biosystems, CA, United States). A plasmid standard (pMD 19-T vector; Takara, Dalian, China) was generated from cloned 16S rRNA genes from the *Ralstonia solanacearum* strain QL-Rs1115 for quantification of bacterial 16S rRNA gene copy numbers.

### Analysis of metagenomic data

#### Metagenomic sequencing, quality control, resistome profiling, and contig assembly

Metagenomic shotgun sequencing libraries were prepared and subsequently sequenced at Shanghai Biozeron Biological Technology Co. Ltd. The sequencing process was conducted utilizing the HiSeq X Ten System (Illumina) in pair-end 150 bp (PE150) mode, and the entire procedure was executed at Shanghai Biozeron Biological Technology Co. Ltd. (Shanghai, China). Fastp (Chen et al., 2018) was used with the default setting to remove adaptors, contaminants, and low-quality reads in raw reads. Filtered reads were assembled with SOAPdenovo2 [22], and obtained contigs were used for binning procedures. The profiling of soil resistome was performed with the ARGs online analysis pipeline (ARGs-OAP) pipeline [23] with the default cut-off values (25 amino acid length, e-value of 1e-07, and 80% identity) for ARG assignment.

### Metagenomic quantification of protist: prokaryote ratio

The protist: prokaryote ratio was used in this study to indicate the relative abundance of protists by considering the sequencing depth of metagenomic data as well as bacterial abundance. The ratio was calculated by the ratio between reads numbers of 18S rRNA genes targeting protists and 16S rRNA genes targeting prokaryotes. We extracted reads targeting rRNA genes using methods modified from previous studies [24–27]. Greengenes2 database [28] and the PR2 database (v.5.0.0) [29] were indexed and used for 16S and 18S rRNA read extraction, respectively. Filtered metagenomic fastq reads were firstly aligned by SortMeRNA [30] against the indexed databases to extract rRNA reads with parameters: -blast “1 cigar qcov qstrand”. USEARCH [31] fastq_filter command was then employed to filter out low-quality potential rRNA reads. Since extracted rRNA gene sequences may come from different rRNA regions (e.g. V4 and V9 regions of 18S rRNA gene sequences of protists), we then implemented the closed-reference workflow, a database-dependent approach utilizing a predefined set of reference sequences to assign taxonomic composition and generate comparable feature tables. In brief, the USEARCH closed_ref command (sequence identity ≥97%) was used to map extracted 16S rRNA and 18S rRNA gene reads against the Greengenes2 and PR2 databases, respectively. Reads assigned to “Mitochondria”, “Chloroplast”, “Opisthokonta”, and “Archaeplastida” were removed from downstream analysis.

### Binning methods, metagenome-assembled genomes quality assessment, and quantification

To explore the correlation between protists and prokaryotic lineages with antibiotic production or resistance capabilities, we first recovered MAGs from metagenomic data. Contigs were binned utilizing MaxBin2 (v2.2.5) [32], MetaBAT2 (v2.12.1) [33], CONCOCT (v1.1.0) [34], and SemiBin (v1.5.1) [35]. Subsequently, the DAS Tool [36] was used to integrate MAGs obtained in each sample through different tools. The completeness and contamination levels of MAGs were evaluated using CheckM [37] with the lineage_wf function. MAGs meeting medium- to high-quality criteria (completeness ≥50% and contamination ≤10%) were selected for further analysis [38].

Filtered MAGs from different samples were subjected to derelictions through the fastANI algorithm in dRep [39], employing a 99% average nucleotide identity (ANI) threshold at the strain level and a 25% between genomes overlap threshold. After derelictions, the remaining MAGs underwent taxonomic annotation using Genome Taxonomy Database (GTDB)-Tk [40] (http://gtdb.ecogenomic.org/) with the GTDB (release 207) as the reference. The relative abundance of these MAGs in each sample was measured via the CoverM pipeline (https://github.com/wwood/CoverM) using the “fpkm” normalization method (fragment per kilobase of transcript per million mapped reads) with the following parameters: “-p bwa-mem --min-read-percent-identity 95 --min-read-aligned-percent 90 -m rpkm”.

### Identification of antibiotic resistance genes and biosynthetic gene cluster in metagenome-assembled genomes

Open reading frames (ORFs) within MAGs were predicted using Prodigal (v.2.6) [41] with the “meta” mode. ARGs were identified by two methods. Firstly, the predicted ORFs were annotated against SARG v3.2-L using BLASTp with DIAMOND [23] employing a sensitive model with an e-value of 1e-5 and identity cut-offs of 70% [42]. Then, a resistance gene identifier (v.6.0.3) with the Comprehensive Antibiotic Resistance Database (CARD, v.3.2.4) [43] as a reference was used to annotate other ORFs, with an identity cutoff of 40%.

The BGCs in each MAG were identified by antiSMASH (v.6.0) [44] with parameters as follows: –taxon bacteria, −genefinding-tool prodigal, −cbknownclusters, and –cc-mibig and –fullhmmer. Clustering analysis of these extracted BGCs was performed using BiG-SCAPE (v.1.1.0, https://github.com/medema-group/BiG-SCAPE) with default settings. The BGCs from antiSMASH analysis, together with previously characterized and clustered BGCs from the MiBiG database (v.2.0) [45], were used as input for Biosynthetic Gene Similarity Clustering and Prospecting Engine (BiG-SCAPE). All BGCs were clustered into gene cluster families based on the similarity network of BGC sequences with a score cut-off of c = 0.7. A sequence similarity matrix of BGCs was obtained from the BiG-SCAPE analysis to construct a network of BGCs, and the network of BGCs was visualized utilizing the ggraph package in R [46]. The nucleotide sequences of each BGC were extracted from the output file generated by antiSMASH. Subsequently, the biological activities of the corresponding secondary metabolites were predicted employing a machine learning model that incorporated the results from three classifiers, namely logistic regression classifier, SVM classifier, and random forest classifiers [47]. BGCs were categorized as having antibiotic activities if at least two out of the three prediction models yielded a probability exceeding 50% for antibacterial or antifungal activity.

### *In vitro* validation of the influence of protists on bacteria communities

The *Naegleria* genus are important phagocytic protists and display a worldwide distribution in soils and plant rhizosphere [48–51]. Members of the *Naegleria* genus have been widely used to study the interplay between bacteria and protists [48,52,53]. Free-living amoeba *Naegleria* sp*.* were cultivated in Page’s amoeba saline (PAS) buffer (prepared by dissolving 136 mg KH_2_PO_4_, 142 mg Na_2_HPO_4_, 120 mg NaCl, 4 mg MgSO_4_·7H_2_O, and 4 mg CaCl_2_·2H_2_O in 1 L of sterile distilled water) with *Escherichia coli* DH5α at a final concentration of 10^5^ CFU mL^−1^ as food resources.

The seven-species synthetic communities (SynComs) were established according to Sun et al. with modification [54]. In short, the initial inoculum for each bacterial species was prepared by culturing overnight in Tryptic Soy Broth (TSB) medium, followed by centrifugation (5000 g, 2 min), and resuspension in TSB medium to achieve an optical density at 600 nm of one (OD_600_ ~ 1). The initial inoculum of SynComs was prepared by mixing equal volumes (20 μL) of the initial inoculum from a single species in 2 mL of 1/2 Msgg+1/2 TSB medium. For co-culture treatment, *Naegleria* sp*.* (1000 cells per mL) were inoculated with the SynComs. Four biological replicates were prepared for each treatment.

### Metatranscriptomic analysis for liquid co-culture experiment

We performed metatranscriptomic analysis to investigate the functional response of SynComs to protist predation. Briefly, after 48 h of co-culturing SynComs with *Naegleria* sp., the culture systems were collected and underwent high-speed centrifugation (10 000 rpm, 1 min) to remove the supernatant. Cell pellets were rapidly frozen in liquid nitrogen and stored at −80°C. Total RNA was extracted using the RNA power soil total RNA isolation kit (MoBio Laboratories, Carlsbad, CA, United States) according to the kit’s instructions and then was sent to Personalbio Genomic Technology Co., Ltd. for library construction and metatranscriptomics sequencing library construction and metatranscriptomic sequencing using the NovaSeq 6000 System (Illumina) with pair-end 150 bp (PE150) mode. Fastp [55] was used with the default setting to perform quality control on raw metatranscriptomics data, and SortMeRNA [30] was used to remove the rRNA gene from filtered fastq data. Transcripts per million (TPM) values of the transcripts were calculated using the tool rsem-calculate-expression [56].

### Statistical analysis

We used principal coordinate analysis based on the Bray–Curtis distance to compare the differences between bacterial and protistan community compositions at the species level in R (v.4.0.1). Bray–Curtis distances were calculated using the “vegdist” function from the vegan package [57] based on the relative taxa abundance matrix. Permutational multivariate analysis of variance test was then performed to investigate significant differences in microbial community composition between different times in soils. ARGs diversities were measured by the number of observed ARGs in each sample. We performed the Wilcoxon tests, if not otherwise specified, to compare different variables between treatments using the “Wilcox. test” function in R. Additionally, we tested linear relationships between the abundance of each MAGs and protistan predation pressure by the “lm” function in R, and the coefficient (ρ) of linear regression was used to reflect the response of MAGs to the relative abundance of protist. We used co-occurrence networks to uncover the potential trilateral relationships between protistan abundance, bacterial abundance, and abundance of each ARG. A Spearman correlation matrix was calculated with the “rcorr” function in the package “Hmisc” in R. The *P*-values were adjusted with the false discovery rate method [58]. Spearman’s correlation coefficient (ρ) higher than 0.6 or lower than −0.6 with *P* < .05 was selected for the construction of trilateral networks.

## Results

### Relationship between protists and antibiotic resistance genes

To investigate the dynamic changes in the relative abundance of protists, abundance of ARGs, and their potential correlations across plant growth, we collected rhizosphere soil samples of tomato plants in week 0, 3, 4, 5, and 6 after tomato planting [19, 59]. We obtained the abundance as well as the richness of ARGs and the relative abundance of soil protists through metagenomic analysis.

We quantified the 16S rRNA copy numbers of all 40 samples using qPCR and examined its linear relationship with the 16S rRNA gene read numbers obtained through metagenomics. The two methods demonstrated a significant concordance (Fig. S1, linear model analysis: explaining 13% of the observed variation, *P* = .029), affirming the reliability of metagenomics-based approaches for quantifying bacterial abundance in the samples. Protist 18S rRNA gene read numbers were likewise measured. The relative abundance of protists that is quantified by the relative abundance of protists and the abundance of ARGs exhibited similar temporal dynamics from the beginning to the end of the experiment (Fig. S2). The compositional structure of resistomes, bacterial, and protistan communities differed among time points (Fig. S3).

**Figure 1:**
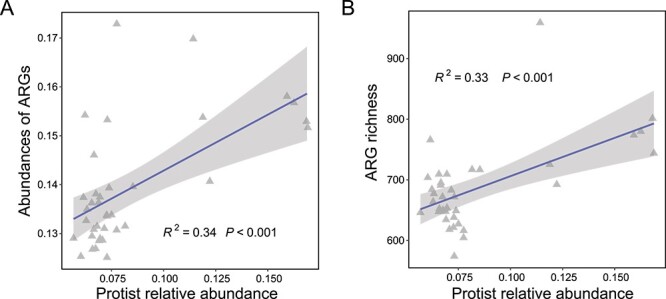
Association between soil protist relative abundance and the abundance and richness of antibiotic resistance genes. Protist relative abundance was defined as normalized abundance (protist rRNA gene copies/bacterial rRNA gene copies). The abundance of antibiotics resistance genes was normalized by bacterial cell numbers (ARG copies per cell). Solid lines represent statistically significant relationships (*P* < .05), as determined by linear regression.

We assessed the contribution of protist communities to soil resistomes. Our linear regression analysis demonstrated that there is a significant positive relationship between the relative abundance of protists and that of ARGs (Fig. 1A, linear regression model analysis: explaining 34% of the observed variation, *P* < .001) and elevated the richness of ARGs (Fig. 1B, linear regression model analysis: explaining 33% of the observed variation, *P* < .001). In particular, the abundance of ARGs belonging to 10 ARG types positively correlated with the relative abundance of protists, while ARGs encoding rifamycin resistance decreased as the relative abundance of protists increased (Fig. S4). Co-occurrence network analysis further explored the relationships among protists, ARGs, and prokaryotes (Figs S5 and S6). The network constructed revealed significant associations between the read numbers of 107 prokaryotic and 25 protistan species with ARG abundance. The network analysis revealed positive connections between phagotrophic protistan species with enriched ARGs (in total, 40 out of 105 positive edges), and these phagotrophic protistan species accounted for, on average, 15.52% of all protist reads recovered by metagenomic data.

### Functional profiling of metagenome-assembled genomes

**Figure 2:**
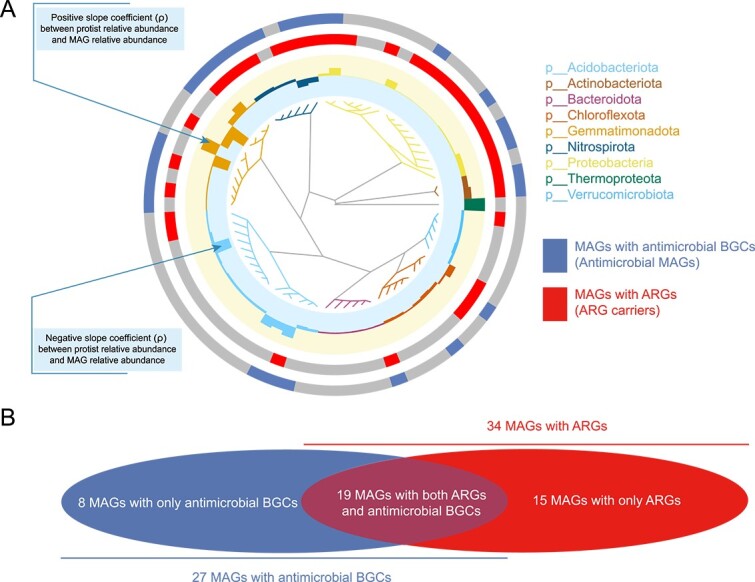
Overview of the MAGs recovered from metagenomic data. The outer ring of the phylogenetic tree shows the coefficients from linear regression analysis between the relative abundance of protists and each MAGs. Branch colors correspond to the phyla of each MAG. The third and fourth rings indicate the presence of antibiotic resistance genes and antibiotic BGCs, respectively. The Venn diagram illustrates that 19 MAGs contain both antibiotic resistance genes and antibiotic BGCs.

To test the correlation between protists and ARGs as well as specific prokaryotic lineages, we retrieved MAGs from metagenomic data and conducted functional profiling on these MAGs. A total of 72 non-redundant prokaryotic genomes were assembled, comprising 71 bacterial MAGs and one archaeal MAG, representing 9 distinct prokaryotic phyla (Table S1). The majority of these MAGs were affiliated with the *Proteobacteria* (17 genomes, 23.6%), followed by *Acidobacteriota* (16 genomes, 22.2%), and *Gemmatimonadota* (11 genomes, 15.3%). Within these MAGs (Fig. 2A), we identified 59 ARGs across 34 MAGs (Table S2 and Fig. S8) and 53 of 315 BGCs associated with potential antibiotic secondary metabolites in 27 MAGs (Fig. 2B and Table S3). The ARGs predominantly fell within the functional categories of efflux pumps (41 ARGs, 69.5%) and antibiotic deactivation mechanisms (5 ARGs, 8.5%), with their distribution primarily observed in *Proteobacteria* (29 ARGs in 14 MAGs), *Nitrospirota* (9 ARGs in 5 MAGs), and *Gemmatimonadota* (7 ARGs in 5 MAGs).

Using BiG-SCAPE for functional classification of BGCs, we predicted that 50.85% of the antibiotic BGCs were linked to ribosomally-synthesized and post-translationally–modified peptides (*n* = 30), 18.64% to terpenes (*n* = 11), 3.39% to polyketide synthases (n = 2), 1.7% to non-ribosomal peptide synthetases (*n* = 1), and 15.3% to other classes (*n* = 9; Fig. S7 and Table S3). The distribution of MAGs harboring these antibiotic BGCs included 34.0% *Gemmatimonadota*, 24.5% *Nitrospirota*, 17.0% *Proteobacteria*, 9.4% *Acidobacteriota*, 9.4% *Actinobacteriota*, 3.8% *Chloroflexota*, and 1.9% *Bacteroidota*. Furthermore, only 19 MAGs contained both ARGs and antibiotic BGCs, with distribution as follows: 6 *Proteobacteria*, 4 *Gemmatimonadota*, 4 *Nitrospirota*, 2 *Actinobacteriota*, 1 *Acidobacteriota*, 1 *Bacteroidota*, and 1 *Chloroflexota* (Fig. 2A and B).

For each MAG recovered from metagenomic data, we evaluated the correlation between their relative abundance with the relative abundance of protists via linear regression models (Fig. 2A, Table S4). Of the 72 MAGs, the relative abundances of 65 genomes exhibited a significant correlation with the relative abundance of protist (Table S4, Linear model analysis: *P* < .05). Among these, relative abundances of 29 genomes positively correlated to the relative abundance of protists, including 11 *Proteobacteria*, 4 *Nitrospirota*, 3 *Gemmatimonadota*, 3 *Acidobacteriota*, 2 *Chloroflexota*, 2 *Actinobacteriota*, and 1 *Thermoproteota*. The remaining MAGs were found to be negatively associated with protist abundance, comprising 13 *Acidobacteriota*, 7 *Gemmatimonadota*, 6 *Bacteroidota*, 6 *Chloroflexota*, 5 *Verrucomicrobiota*, and 2 *Nitrospirota*.

### Bacterial antibiotic biosynthesis and antibiotic resistance

**Figure 3:**
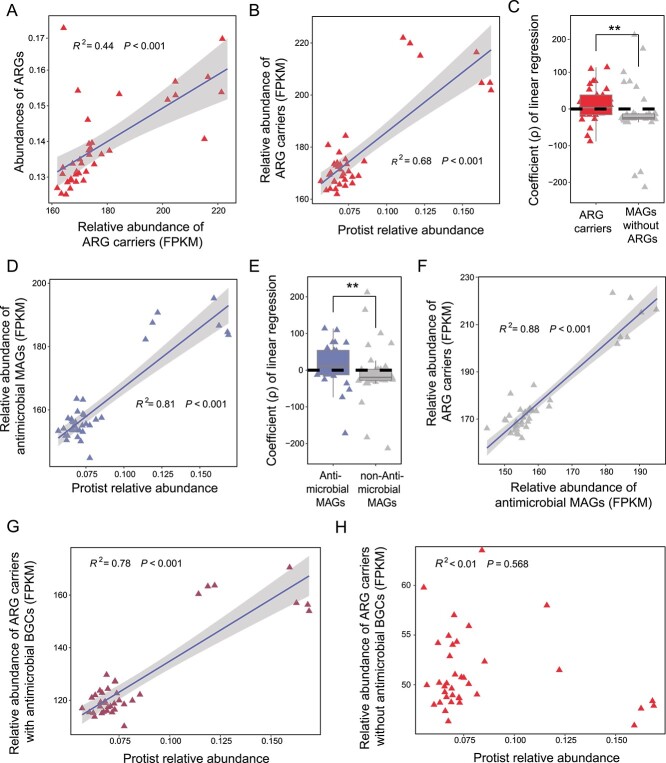
MAGs carrying antibiotic resistance genes and BGCs predict community-level antibiotic resistance gene accumulation. Correlations between the abundance of MAGs carrying antibiotic resistance genes (ARGs) and overall ARG abundance were depicted in panel (A). Significant linear relationships were observed between the relative abundance of protists with the abundance of ARG carriers (B) and the abundance of MAGs with antibiotic BGCs (D). Both ARG carriers (C) and MAGs with antibiotic BGCs (E) exhibited potentially higher resistance to protists. The abundance of ARG carriers with antibiotic BGCs positively correlated with the relative abundance of protists (F). ARGs without antibiotic BGCs did not show a significant linear relationship with the relative abundance of protist (H). In panels (A), (B), (D), (F), (G), and (H), solid lines indicate statistically significant relationships (*P* < .05) based on linear regression models, while dashed lines indicate non-significant relationships (*P* > .05). Statistical significance in panels (D) and (F) is determined by Wilcoxon tests (NS = non-significant; * = *P* < .05; ** = *P* < .01; *** = *P* < .001).

We investigated the role of ARG carriers in the accumulation of ARGs in soils, finding that the relative abundance of ARG carriers demonstrated substantial explanatory power for the overall abundance of ARGs in soils (Fig. 3A, linear regression model analysis: explaining 44% of the observed variation, *P* < .001). Furthermore, the relative abundance of ARG carriers positively correlated with the relative abundance of protists (Fig. 3B, linear regression model analysis: explaining 68% of the observed variation, *P* < .001). Moreover, compared to MAGs carrying no antibiotic resistance gene, ARG carriers potentially showed higher resistance to protists revealed by coefficients of linear regression analysis between the relative abundance of protists and the relative abundance of each MAG (Fig. 3C, Wilcoxon tests, *P* = .008). Conversely, the overall abundance of all 72 MAGs did not show a significant linear relationship with the relative abundance of protist (Fig. S9A, linear regression model analysis: *P* = .86) and negatively correlated with the overall abundance of ARGs (Fig. S9B, linear regression model analysis: explaining 10% of the observed variation, *P* = .029). These findings underscore the important contribution of the relative abundance of ARG carriers to the overall abundance of ARGs in soils.

The relative abundance of protists showed greater explanatory power (81% vs. 68%, Fig. 3D) for the abundance of antibiotic BGC carriers compared to that of ARG carriers. Antibiotic BGC carriers also demonstrated significantly higher resistance to protists than non-toxic counterparts (Fig. 3E, Wilcoxon tests, *P* = .007). Given the significant linear relationship between the abundances of antibiotic BGC carriers and ARG carriers (Fig. 3F, linear regression model analysis: explaining 88% of the observed variation, *P* < .001), and the fact that most ARG carriers also contained antibiotic BGCs, our subsequent focus was on the importance of antibiotic BGCs in ARG carriers in resisting protist predation. Among ARG carriers, only those with antibiotic BGCs positively correlated with the relative abundance of protists (Fig. 3G, linear regression model analysis: explaining 78% of the observed variation, *P* < .001), whereas ARG carriers without antibiotic BGCs did not show such a significant correlation (Fig. 3H, linear regression model analysis: *P* = .57). Although both types of ARG carriers contributed to the increased abundance of ARGs in soils (Fig. S10A and B), those with antibiotic BGCs showed a higher contribution (32.6% vs. 19.7%, Fig. S10C).

We observed potential collective antibiotic resistance between ARG carriers capable of deactivating antibiotics and antibiotic BGC carriers having no ARGs (Fig. S11A, linear regression model analysis: explaining 87% of the observed variation, *P* < .001). However, this cooperative antibiotic resistance might compromise the resistance of ARG carriers against protists, as their abundance decreased with increasing relative abundance of protists (Fig. S11B, linear regression model analysis: explaining 69% of the observed variation, *P* < .001).

### Validation of the influence of protists on bacteria communities

To investigate the influence of protist predation on the expression of ARGs abundance within bacterial communities, we incubated a free-living amoeba (*Naegleria* sp.) with bacterial SynComs together. The SynComs included the six stable bacterial species of *Acinetobacter baumannii* XL380, *Chryseobacterium rhizoplanae* XL97, *Comamonas odontotermitis* WLL, *Enterobacter bugandensi*s XL95, *Pantoea eucrina* XL123, and *Pseudomonas stutzeri* XL272 [54] together *Bacillus velezensis* SQR9, a bacteria strain known for producing antibiotics [60, 61] and harboring multiple ARGs [62].

**Figure 4:**
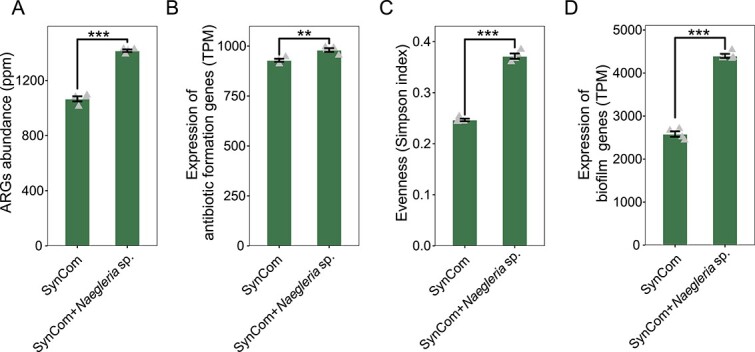
Microcosm experiment showing the impacts of protist grazing on the bacterial communities. The effect of protistan predator *Naegleria* sp. on the expressions of antibiotic resistance genes (A), antibiotics biosynthesis genes (B), the evenness (C), and biofilm-related genes (D) of bacterial communities for gene expression in microcosm. In each panel, statistical significances between treatments were determined by the *t*-tests (NS, non-significant; *P < .05; **P < .01; ***P < .001).

Co-incubation with *Naegleria* sp. led to a significant increase in the expression of ARGs of bacterial communities (Fig. 4A, *t*-tests, *P* < .001). Additionally, the expression of genes encoding antibiotic secondary metabolites was also upregulated in the presence of predatory protists (Fig. 4B, *t*-tests, *P* = .006; Table S5). In addition, the expression of ARGs and genes encoding antibiotics showed a significantly strong linear relationship (Fig. S12, linear regression model analysis: explaining 71% of the observed variation, *P* < .05), indicating that the increased ARGs abundances causally resulted from accumulations of antibiotics produced to counter protist invasion. Moreover, we used the RNA Shannon index to measure the uniformity of the bacterial community [63, 64]. Our analysis found that co-incubation with *Naegleria* sp. increased the uniformity of bacterial communities (Fig. 4C, *t*-tests, *P* < .001). In particular, co-incubation with *Naegleria* sp. sustained the level of *B. velezensis* SQR9 (from 30 TPM to 18 750 TPM) in bacterial communities. Furthermore, we also found other defensive mechanisms were activated by bacterial communities to fight against protist predation, as evidenced by the upregulated expression of genes associated with biofilm formation during co-incubation with *Naegleria* sp. (Fig. 4D, *t*-tests, *P* < .001; Table S6).

**Figure 5:**
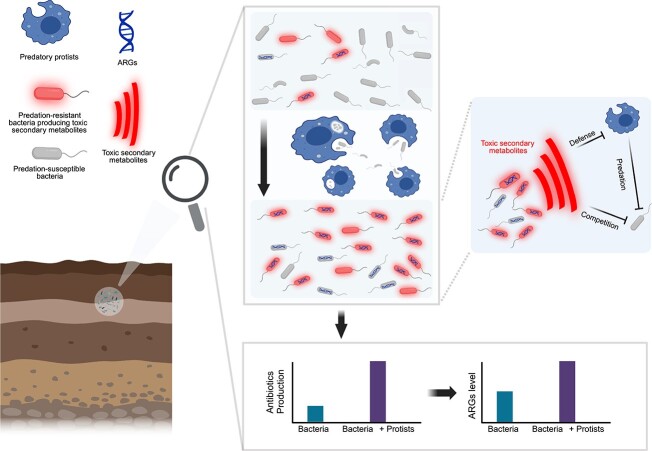
Conceptual model depicting how protist predation promotes the spread of antimicrobial resistance by fostering antagonistic interactions in the microbiome. In soil ecosystems, antibiotics-producing bacteria (APB) and non-APB coexist. APB produce antibiotics to gain a competitive advantage against competitors or to combat predators. APB often harbor ARGs as a defense mechanism against their antibiotic compounds, making them ARG carriers. Selective grazing by protists promotes the proliferation of APB, thereby increasing the abundance of ARGs in soil environments.

## Discussion

In this study, we investigate the underlying mechanism by which soil protists could stimulate the accumulation of ARGs. We provide novel evidence that indicates a selective pressure by soil protists on microbial communities that would increase, as a consequence of bacterial community structure change, the abundance of ARGs in both field and laboratory conditions. We found that soil protists induce the enrichment of ARGs by selectively preying on bacteria that produce antibiotics and harbor ARGs.

We observed that antibiotic-producing bacteria (APB) maintained a high relative abundance under conditions of elevated protist abundance, suggesting potentially higher resistance to protist pressures. In our laboratory experiments, the antibiotic-producing *B. velezensis* increased in abundance when co-incubated with protists, while it declined in the absence of protists. These findings collectively supported the hypothesis that selective consumption by protists enriches APB. Producing toxic secondary metabolic compounds is one of the most important strategies for bacteria to fight against protistan grazing [65–67]. For example, certain antibiotic-producing bacterial strains *Janthinobacterium lividum* and *Chromobacterium violaceum* [65], *Pseudomonas fluorescens* [17,18,52,68] have been reported to produce antibiotics to inhibit the growth of protistan predators. Protistan predation could also promote the prevalence of APB. For instance, antibiotic-producing *P. fluorescens* CHA0 gains a competitive advantage in the rhizosphere over its non-antibiotic-producing mutants under protist predation [13]. Consequently, protist-bacteria interactions may favor the proliferation of APB, leading to increased antibiotic production [19, 69].

Antibiotics and ARGs can function as both “weapons” and “shields” within microbial communities [4, 70], necessitating the development of resistance mechanisms by APB to mitigate the adverse effects of their own antibiotics [71]. It is common for antibiotic producers to harbor resistance genes as a self-protection strategy against the compounds they produce [72]. Our metagenomic investigation revealed that APB are predominantly ARG carriers. Non-antibiotic producers also carry ARGs, constituting 15 out of 27 ARG carriers. These ARGs may originate from mutations in pre-existing genes (proto-resistance genes) within the bacterial genome [73–75] or from the gene elements of another organism via horizontal gene transfer (HGT) [76, 77]. Additionally, our study unveils instances where antibiotic producers lost their ARGs but were sustained by ARG carriers equipped with potential antibiotic deactivation mechanisms. This supports the notion of collective cooperative antibiotic resistance, where bacterial communities thrive in the presence of antibiotics, even if individual members may not [78–80]. This again highlights that community-level studies may result in different outcomes compared with species or isolate-level studies, given complex species–species cooperation under different biotic or abiotic stress. In summary, our research underscores that members capable of antibiotic production dominate the pool of ARG carriers.

The presence of protistan predators may lead to higher bacterial population growth and increased mutation rates, thereby driving the evolution of defense mechanisms against antibiotics [81–83]. As demonstrated by a previously published work [84], bacterial prey *P. fluorescens* SBW25 gains greater fitness benefits in response to protistan predation as the levels of antibiotics increase. Furthermore, concurrent selection by antibiotics and bacterial consumers might lead to increased population sizes and the spread of double-resistant bacteria to both protistan predators and antibiotics. Because the consumption of bacteria by protistan predators [85–87] and bacterial lysis induced by antibiotics [88, 89] could release nutrients into the surrounding environment. The double-resistant bacteria can then utilize these nutrients as food sources, thereby promoting their growth and contributing to the overall antibiotic resistance of the bacterial community. This interconnected relationship highlights the intricate ecological dynamics influenced by antibiotics and bacterial consumers in shaping bacterial communities and their resistance profiles.

In addition to the predation of protistan predators, other possible mechanisms that contribute to the increased levels of ARGs have been reported. For example, research in seawater suggests that the consumption of bacteria by ciliates and heterotrophic nanoflagellates leads to the release of extracellular ARGs [90], which can then be dispersed among bacterial communities via natural transformation [91]. It is also important to acknowledge that certain protistan predators can indirectly facilitate the HGT of ARGs among bacterial species within their bodies [92–94]. Additionally, certain protistan predators might potentially carry ARGs and actively transfer them to associated bacteria [95, 96]. In addition to the secretion of secondary metabolites or toxins, bacteria have evolved alternative strategies to survive under protistan grazing [10, 66, 97], such as morphological alteration into inedible forms [98], the development of biofilms [99], and enhancement of motility [100]. These mechanisms account for the positive associations between protistan predation pressures and the expression of certain functional genes in bacteria.

In conclusion, our study demonstrates that soil protists consistently promote the accumulation and expression of ARGs in both synthetic communities and agricultural soils. This effect was potentially mediated by the shift toward more antagonisms among bacterial communities, as evidenced by the enhanced abundance of bacteria capable of producing antibiotics (Fig. 5). Consequently, managing predation by protists may offer valuable leverage to control the spread of antimicrobial resistance in natural environments.

## Supplementary Material

Supplementary_Figures_wrae169

Supporting_Tables_wrae169

## Data Availability

The raw data of metagenomics-derived gene catalogs are publicly available under the accession number PRJNA492172.
