# Supplementary material for: Protist predation promotes antimicrobial resistance spread through antagonistic microbiome interactions
Source: ISME J. 2024 Sep 4;18(1):wrae169. doi: 10.1093/ismejo/wrae169 (PMC11453101; doi:10.1093/ismejo/wrae169)
Supplement: Supplementary_Figures_wrae169 [file supplementary_figures_wrae169.pdf]

Supplementary figures

Title: Protist predation promotes antimicrobial resistance spread through antagonistic microbiome interactions

Running title: Protist and antibiotic resistance spread

Authors: Chen Liu[1], Yijin Wang[1], Zeyuan Zhou[1], Shimei Wang[1], Zhong Wei[1], Mohammadhossein Ravanbakhsh[2], Qirong Shen[1], Wu Xiong[1,*], George A. Kowalchuk[2], and Alexandre Jousset[1,2]

Affiliations:

[1]Jiangsu Provincial Key Lab for Solid Organic Waste Utilization, Key Lab of Organic-Based Fertilizers of China, Jiangsu Collaborative Innovation Center for Solid Organic Wastes, Educational Ministry Engineering Center of Resource-Saving Fertilizers, Nanjing Agricultural University, Nanjing 210095, People's Republic of China

[2]Ecology and Biodiversity Group, Department of Biology, Institute of Environmental Biology, Utrecht University, Padualaan 8, 3584 CH Utrecht, The Netherlands

*To whom correspondence should be addressed: Wu Xiong (wuxiong@njau.edu.cn)

This PDF file includes:

Fig. S1. Relationship between 16S reads revealed in metagenomic data and bacterial population densities measured by qPCR.

Fig. S2. Temporal dynamics of ARGs abundances (a) and protist pressures (b) among six weeks.

Fig. S3. Temporal dynamics of the composition of soil resistomes (a), bacterial community composition (b) and protist community composition (c) in soils alongside the six week

Fig. S4. Significant linear relationships between the ARG abundance of ten ARGs types and protist pressure.

Fig. S5. Co-occurrence network illustrating the association of abundance of each ARG with bacterial and protist genera.

Fig. S6. Taxonomic affiliation of 41 protist nodes (a) and the mechanism for the 200 ARGs nodes in the networks.

Fig. S7. Prediction of BGCs and their potentially associated secondary metabolites.

Fig. S8. Mechanism for 59 ARGs identified in 34 ARGs carriers.

Fig. S9. Relationships between the overall abundance of all MAGs with protist pressure (a) and ARGs abundances (b).

Fig. S10. Linear relationships between ARGs abundances with two types of ARGs carriers (a and b) and the contribution of the two types of ARG carriers to the overall ARG abundances revealed by VPA analysis (c).

Fig. S11. Abundance of MAGs containing enzymatic deactivating ARGs exhibited a positive correlation with MAGs lacking ARGs (a), while displaying a negative correlation with protist pressures (b).

Fig. S12. Relationship between the expression of ARGs and antibiotics encoding genes in lab assay.

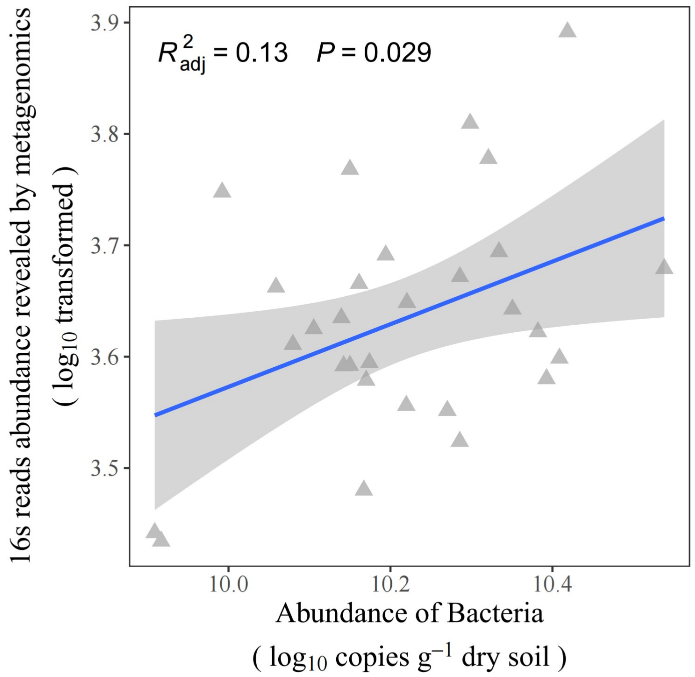

Fig. S1. Relationship between 16S reads revealed in metagenomic data and bacterial population densities measured by qPCR.

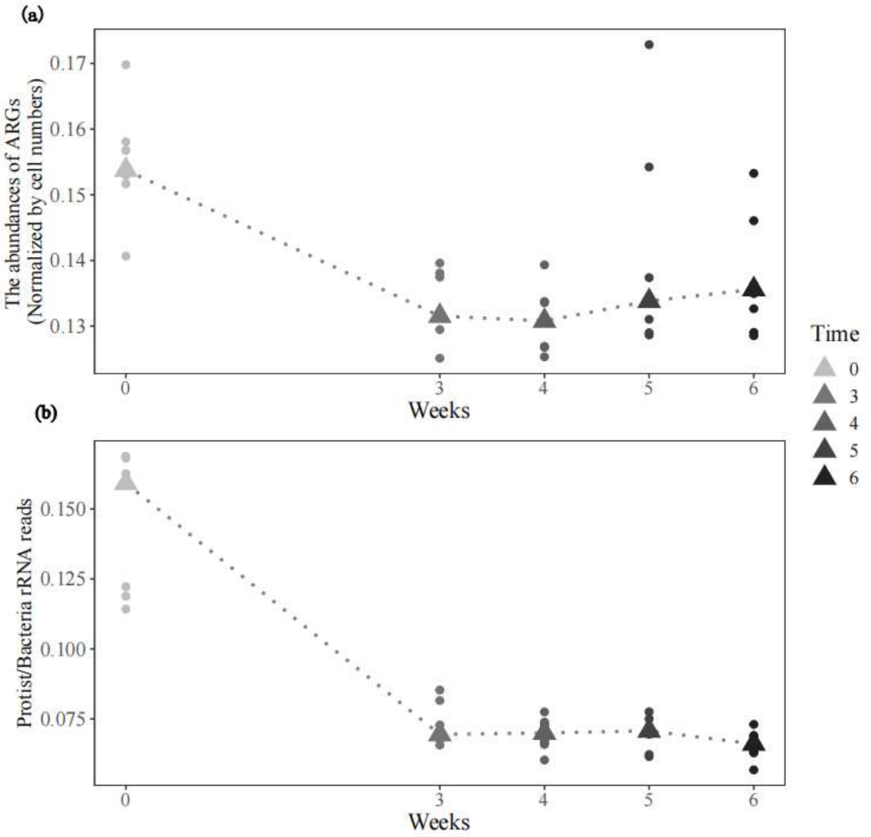

Fig. S2. Temporal dynamics of ARGs abundances (a) and protist pressures (b) among six weeks.

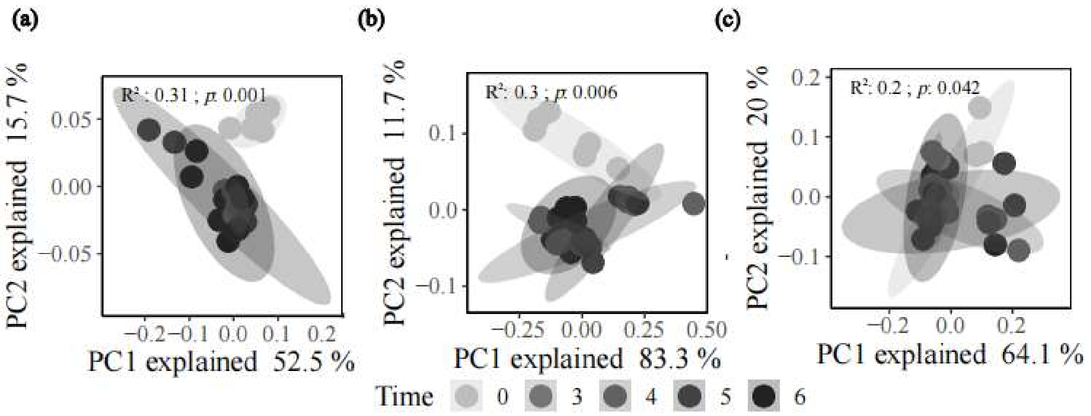

Fig. S3. Temporal dynamics of the composition of soil resistomes (a), bacterial community composition (b) and protist community composition (c) in soils alongside the six week.

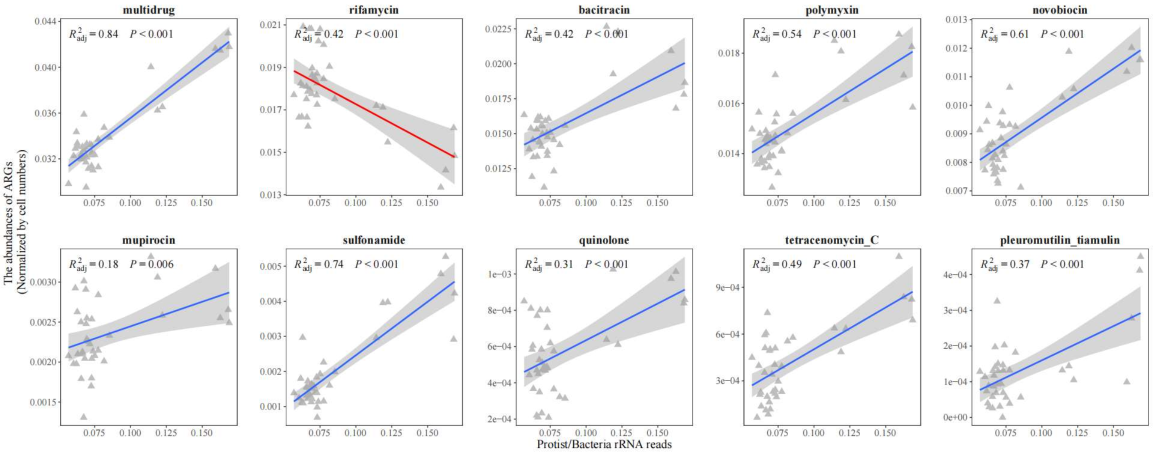

Fig. S4. Significant linear relationships between the ARG abundance of ten ARGs types and protist pressure.

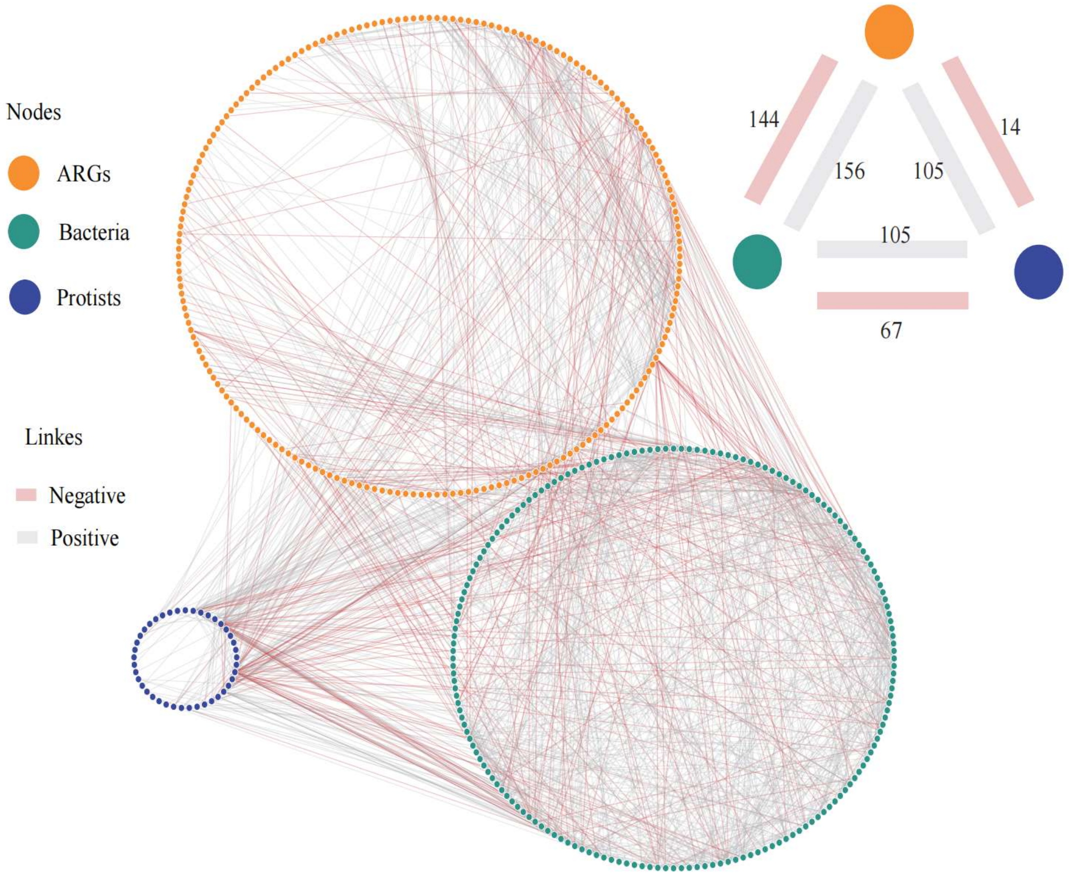

Fig. S5. Co-occurrence network illustrating the association of abundance of each ARG with bacterial and protist genera.

Each node in the network represents a taxon at the species level. The top-right of the network provides a summary of edge statistics. Colored numbers within the network represent the count of nodes belonging to the corresponding category (positive: $\rho \geq 0.6$, $p < 0.05$, in grey; negative: $\rho \leq -0.6$, $p < 0.05$, in red).

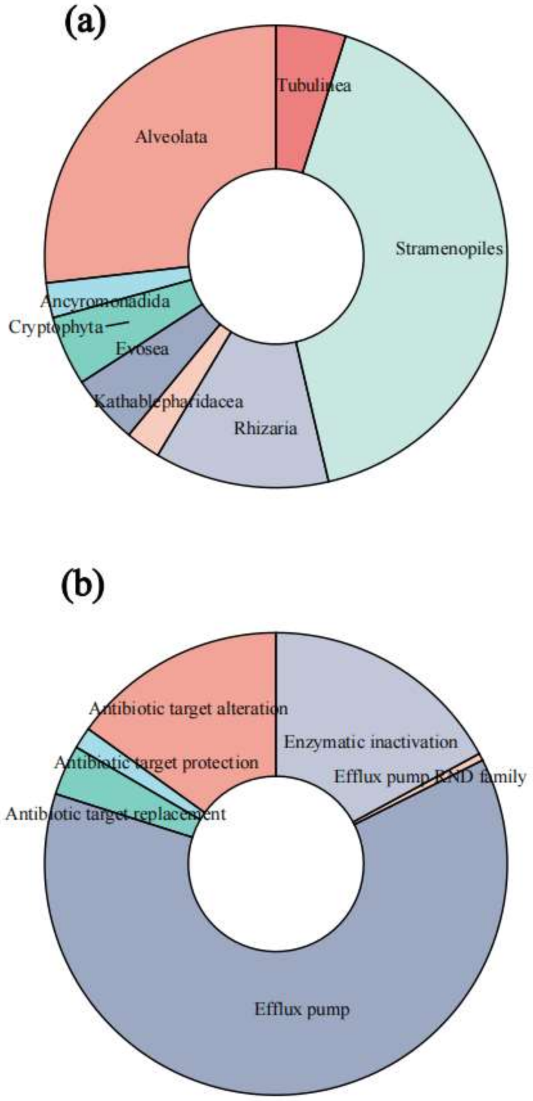

Fig. S6. Taxonomic affiliation of 41 protist nodes (a) and the mechanism for the 200 ARGs nodes in the networks.

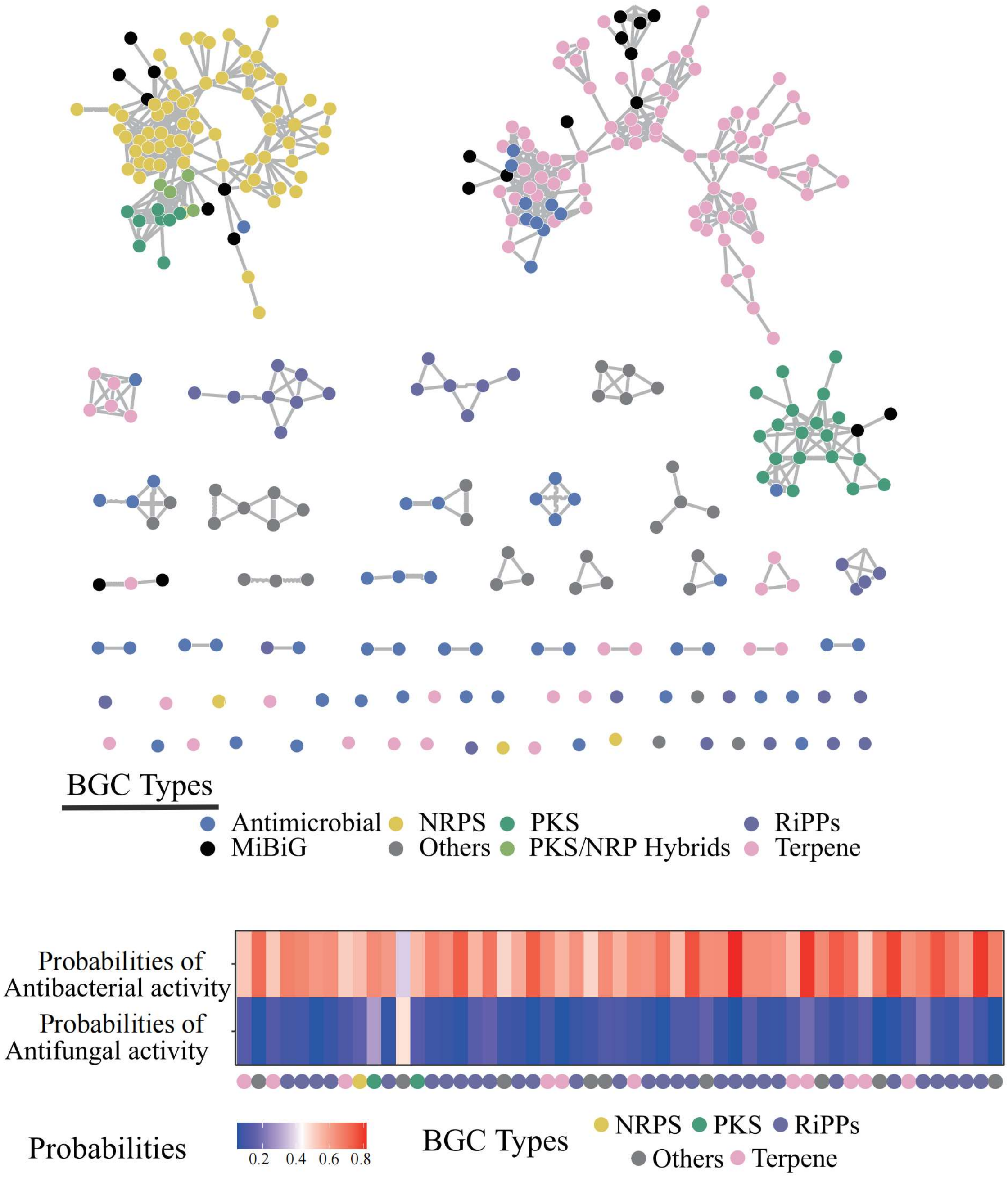

Fig. S7. Prediction of BGCs and their potentially associated secondary metabolites.

A Gene Cluster Family network illustrates the relationships among BGCs in MAGs, with each node representing a distinct BGC. The prediction of biological activities for these BGCs was visualized in panel through a heatmap, depicting the probabilities of different biological activities. Only BGCs with high probabilities of antibacterial activity (>50%) and antifungal activity (>50%) were highlighted in the stacked columns.

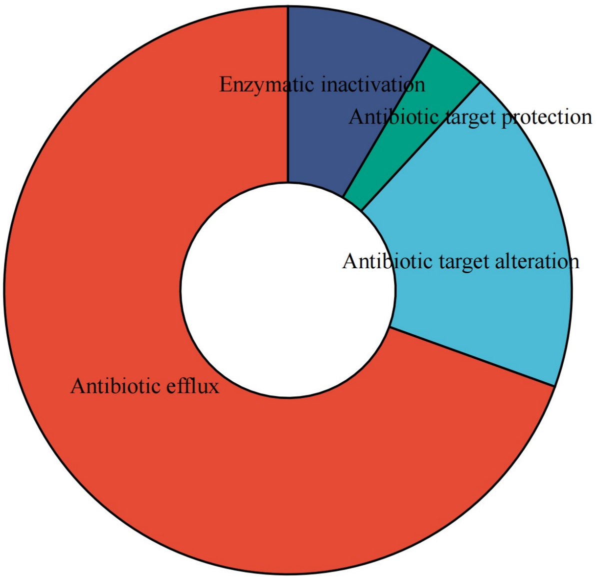

Fig. S8. Mechanism for 59 ARGs identified in 34 ARGs carriers.

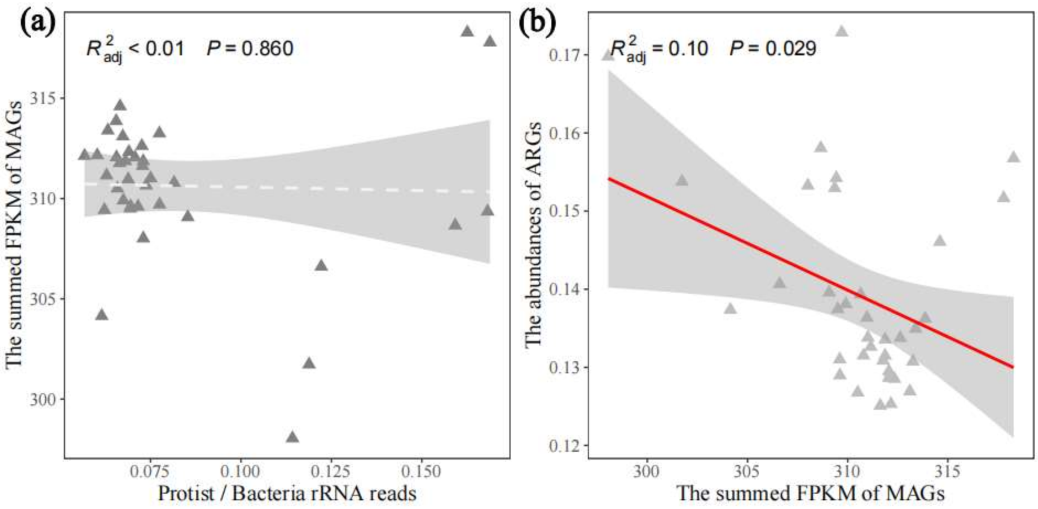

Fig. S9. Relationships between the overall abundance of all MAGs with protist pressure (a) and ARGs abundances (b).

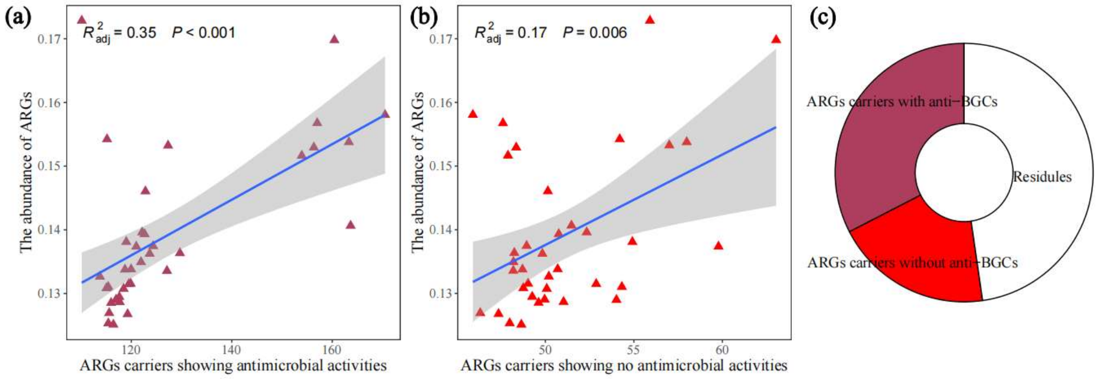

Fig. S10. Linear relationships between ARGs abundances with two types of ARGs carriers (a and b) and the contribution of the two types of ARG carriers to the overall ARG abundances revealed by VPA analysis (c).

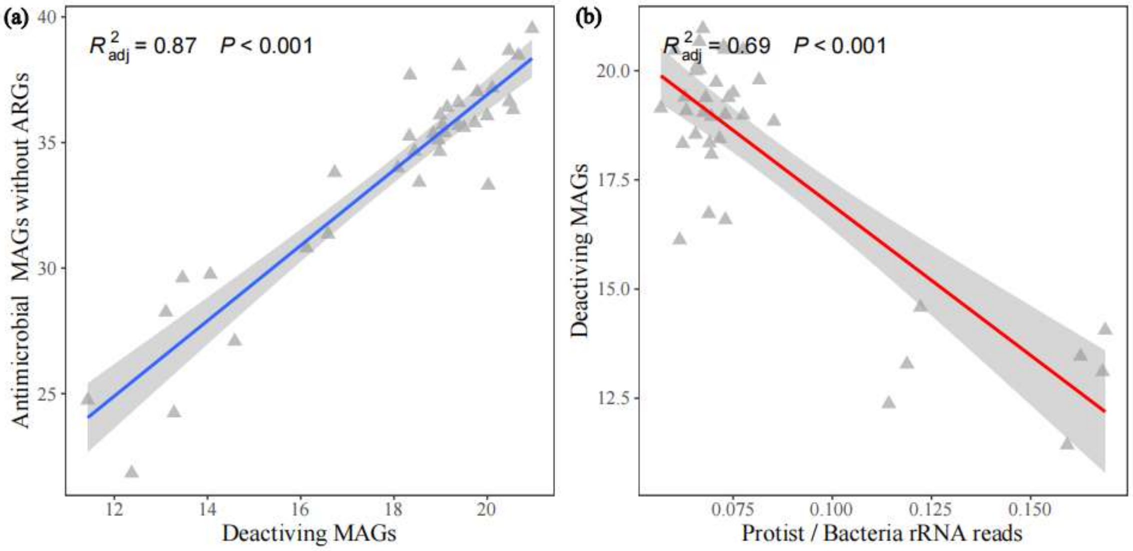

Fig. S11. Abundance of MAGs containing enzymatic deactivating ARGs exhibited a positive correlation with MAGs lacking ARGs (a), while displaying a negative correlation with protist pressures (b).

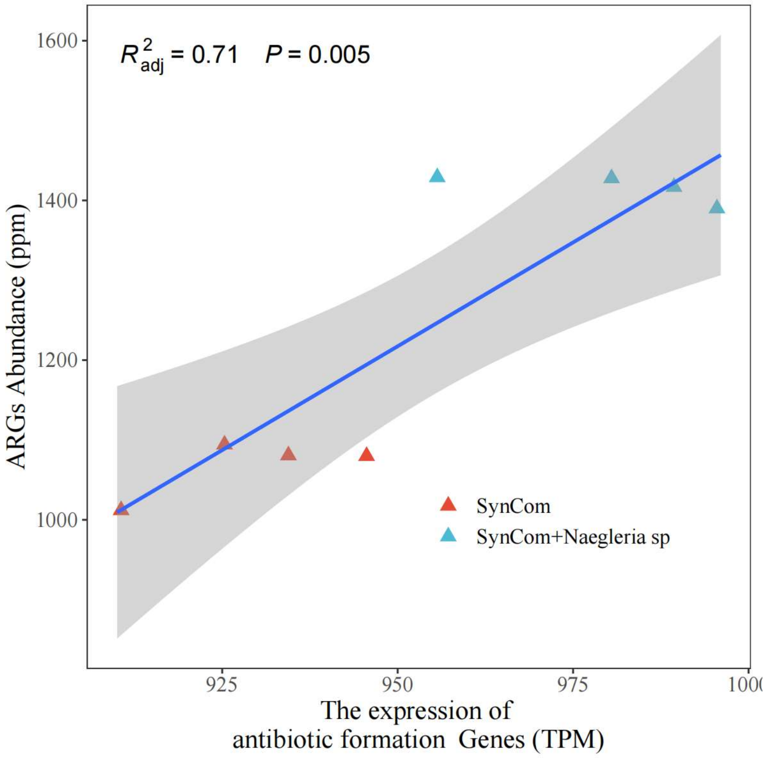

Fig. S12. Relationship between the expression of ARGs and antibiotics encoding genes in lab assay.
